# Design of a Recyclable Photoresponsive Adsorbent via Green Synthesis of Ag Nanoparticles in Porous Aromatic Frameworks for Low-Energy Desulfurization

**DOI:** 10.3390/molecules31020248

**Published:** 2026-01-12

**Authors:** Tiantian Li, Xiaowen Li, Hao Wu, Qunyu Chen

**Affiliations:** School of Chemical Engineering and Technology, Xuzhou College of Industrial Technology, Xuzhou 221140, China; xiaowenli89@163.com (X.L.); wuh@mail.xzcit.cn (H.W.); cqy@mail.xzcit.cn (Q.C.)

**Keywords:** photothermal adsorption, silver nanoparticles, porous aromatic frameworks, desulfurization, stimuli-responsive materials, thiophenic sulfides

## Abstract

Based on the pressing need to develop efficient desulfurization technologies for fuel oils, this study presents a novel photoresponsive adsorbent for the removal of refractory thiophenic sulfides. Conventional hydrodesulfurization exhibits limited efficiency for such compounds, while adsorption–desorption processes often suffer from high energy consumption during regeneration. Inspired by natural stimuli-responsive systems, we designed a photothermal adsorbent by incorporating silver nanoparticles (Ag NPs) into a porous aromatic framework (PAF) via a green photoreduction method. The resulting materials, denoted as Ag(0)PBPAF-n (*n* = 1, 2, 3), were thoroughly characterized to confirm successful synthesis and structural integrity. The introduced Ag NPs serve as adsorption sites, enhancing uptake capacity through weak interactions with sulfur atoms in thiophenic molecules. More significantly, under light irradiation, the localized surface plasmon resonance (LSPR) of Ag NPs enables efficient photothermal conversion, triggering rapid desorption without conventional heating. Adsorption–desorption tests demonstrated that up to 48% of adsorbed thiophenic sulfur could be released upon illumination. Fixed-bed experiments further verified that light can effectively stimulate regeneration and improve energy efficiency. This work offers a promising strategy for designing recyclable adsorbents with low-energy regeneration driven by clean solar energy.

## 1. Introduction

The presence of sulfur compounds in fuel oils leads to the emission of substantial amounts of toxic sulfur oxides (SO_X_) during combustion, causing a series of environmental issues such as haze and acid rain, which significantly impact human health and the ecosystem [1,2,3,4,5,6,7,8]. In response to stringent environmental regulations, there is an urgent need to develop technologies for reducing sulfur content in fuels. Among sulfur species, thiophenic compounds are particularly challenging to remove via conventional hydrodesulfurization (HDS) due to their stable aromatic heterocyclic structure and steric hindrance effects around the sulfur atom [9,10,11,12,13,14]. Adsorptive desulfurization has emerged as a highly competitive alternative, owing to its mild operating conditions and simple equipment requirements [15,16,17,18,19,20,21,22]. However, the efficient regeneration of adsorbents remains a critical factor determining the feasibility of this technology. Traditional regeneration methods, such as pressure-swing and thermal desorption, are often energy-intensive and require complex infrastructure [23,24,25]. Therefore, the development of adsorbents that combine high adsorption capacity with low-energy regeneration capability is of paramount significance.

In nature, numerous organisms can adapt their properties in response to environmental changes. For instance, sunflowers adjust their leaf orientation according to sunlight intensity. Inspired by such biological systems, the development of stimuli-responsive materials has become a promising research field [26]. These materials can alter their physical or chemical properties upon exposure to external stimuli such as temperature [27], pH [28], magnetic fields [29], and light [30]. Among these, light offers several distinct advantages: (1) it can be remotely and easily controlled, (2) it allows for precise regulation, and (3) it is a clean and safe energy source. Consequently, light-responsive materials have attracted significant research interest in recent years. For example, Xu et al. [31] integrated photoresponsive azobenzene–silane groups with aerogels to achieve precise control over the adsorption and desorption of dysprosium ions. A specific subclass of these materials, known as photothermal materials, can convert absorbed light energy into thermal energy under certain conditions [32,33,34]. Compared to conventional heating methods, photothermal materials significantly improve energy utilization efficiency [35,36,37,38]. For adsorption processes, this unique photothermal conversion effect can greatly enhance heat transfer and reduce regeneration costs.

Within the field of adsorption separation, Ag NPs exhibit an LSPR effect under visible light irradiation. When exposed to light, the free electrons in Ag NPs undergo collective oscillation, efficiently converting light energy into heat [39]. This exceptional photothermal property provides a compelling research pathway. Furthermore, Ag NPs exhibit a weak interaction with thiophenic sulfur compounds, strengthening their binding with sulfur atoms and thereby enhancing the adsorption capacity for these species [40,41]. Various methods have been developed for synthesizing Ag NPs, including chemical reduction (e.g., sodium borohydride reduction, electrochemical reduction, thermal decomposition), biological reduction (using bacterial or plant extracts), and physical reduction (e.g., physical vapor condensation, arc discharge). Among these, photoreduction stands out due to its notable advantages: (1) it produces Ag NPs with narrow size distribution, high uniformity, and good dispersity; (2) it avoids the use of highly toxic or reactive chemicals; and (3) it is a simple and mild process. These benefits make photoreduction one of the most favored synthesis methods.

**Scheme 1:**
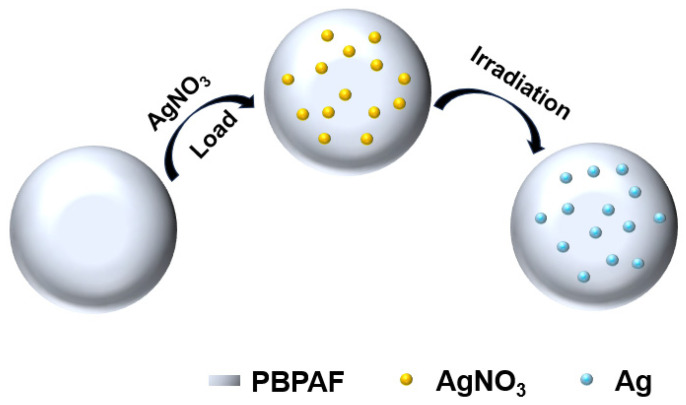
The synthetic process of Ag(0)@PBPAF.

Based on the above considerations, we introduce the concept of photothermal materials into the field of adsorptive desulfurization, aiming to achieve low-energy adsorbent regeneration using clean solar power. In this work, a PAF with high specific surface area was selected as the support to anchor Ag NPs via a green and facile photoreduction strategy. A series of photothermal adsorbents with different Ag loadings, denoted as Ag(0)PBPAF-n (n = 1, 2, 3), were successfully fabricated, in which the Ag NPs act as both adsorption and photothermal desorption sites (Scheme 1). The well-defined structure and successful formation of the composites were confirmed by a suite of characterization techniques. Experimental results demonstrated that the incorporated Ag(0) sites significantly enhanced the adsorption capacity of the pristine PBPAF support, which is attributed to the specific weak interaction between Ag NPs and sulfur atoms in thiophenic compounds. More importantly, upon visible light irradiation, the photothermal effect of Ag NPs enabled rapid and efficient desorption, with up to 48% of the adsorbed thiophenic sulfur being released. Fixed-bed column experiments further verified that light-triggered desorption not only achieved high efficiency but also substantially reduced energy consumption compared to conventional thermal regeneration. This study designs a bifunctional photothermal adsorbent that integrates selective adsorption and light-driven regeneration into one material system, providing a novel and energy-efficient strategy for advanced desulfurization processes.

## 2. Results and Discussion

### 2.1. Structure and Surface Properties of Ag(0)@PBPAF Composites

**Figure 1:**
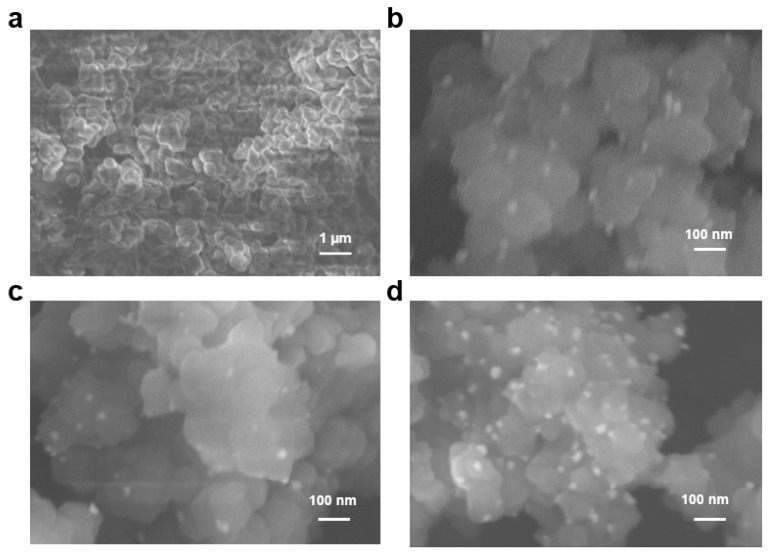
SEM images of (**a**) PBPAF, (**b**) Ag(0)@PBPAF-1, (**c**) Ag(0)@PBPAF-2, (**d**) Ag(0)@PBPAF-3.

To investigate the structural stability of the PBPAF material after the incorporation of Ag nanoparticles, scanning electron microscopy (SEM) was first employed. As shown in Figure 1a, the pristine PBPAF material consists of uniform spherical particles with an average diameter of approximately 200 nm. After loading with different amounts of Ag nanoparticles, the resulting Ag(0)@PBPAF composites well maintain this spherical morphology without significant changes in particle size or shape (Figure 1b–d). Furthermore, the SEM images clearly confirm the successful deposition of Ag nanoparticles onto the PBPAF matrix.

**Figure 2:**
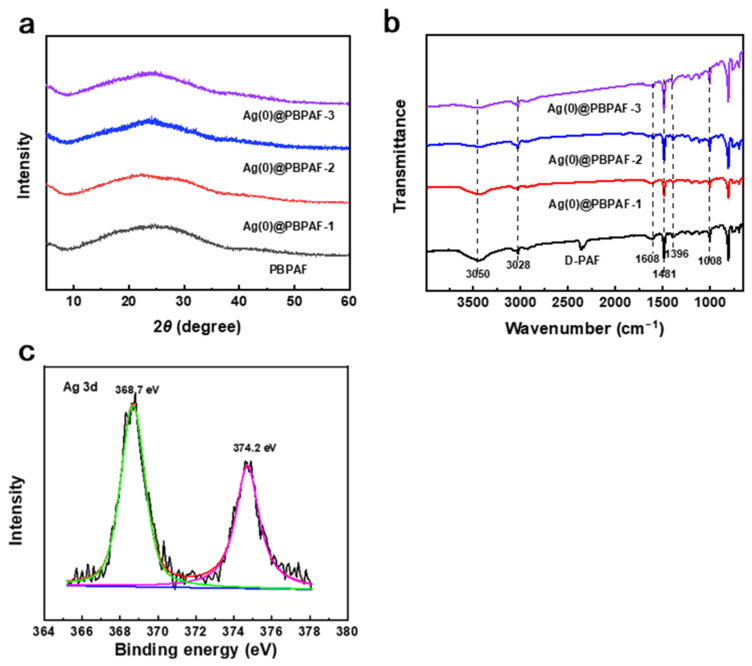
(**a**) Wide-angle XRD map, (**b**) FT-IR map of PBPAF and Ag(0)@PBPAF, (**c**) Ag 3d orbital spectrum of Ag(0)@PBPAF-2.

The crystallinity of the samples was examined by X-ray diffraction (XRD). As displayed in Figure 2a, the PBPAF material shows no distinct diffraction peaks within the 2θ range of 5–60°, indicating its amorphous nature, which is consistent with the SEM observations. The XRD patterns of the Ag(0)@PBPAF composites are like those of the bare PBPAF, suggesting that the introduction of Ag nanoparticles did not alter the host framework. Notably, no characteristic diffraction peaks corresponding to crystalline Ag phases are detected, which is likely due to the low loading amount and high dispersion of Ag nanoparticles within the porous support [42]. Fourier transform infrared (FT-IR) spectroscopy was further conducted to analyze the chemical structures. As presented in Figure 2b, the spectrum of PBPAF shows characteristic absorption bands in the region of 3020–3040 cm^−1^, which may contain contributions from vibrations and O–H stretching from residual adsorbed moisture. The more distinct and consistent bands at 1481 and 1396 cm^−1^ (characteristic skeletal vibrations of the aromatic ring) and 1008 cm^−1^ (aromatic C–C and C–H bending vibrations) confirm the integrity of the aromatic framework. The presence of a minor band near 1608 cm^−1^ across all samples corroborates the presence of some adsorbed water. The FT-IR spectra of the Ag(0)@PBPAF composites are nearly identical to that of the parent PBPAF, indicating no significant chemical alteration of the framework after Ag incorporation. Moreover, the absence of absorption bands at approximately 535 cm^−1^ and 1400 cm^−1^, which are typically associated with Ag_2_O, confirms that the silver species exist predominantly in their metallic state (Ag(0)). Furthermore, the broad absorption band centered around 3450 cm^−1^, attributable to O–H stretching vibrations from surface-adsorbed water, is observed to gradually weaken in the Ag(0)@PBPAF composites with higher Ag loading (Figure 2b). This change can be explained by two factors: (i) the increasing occupancy of the porous network by Ag nanoparticles may physically reduce the available surface area and pore volume for water physisorption, as supported by the N_2_ sorption data (Table 1); and (ii) the incorporation of relatively hydrophobic metallic Ag sites may alter the overall surface hydrophilicity of the composite, making it less prone to adsorb atmospheric moisture compared to the pristine organic framework. These collective findings demonstrate the structural stability of the composite during the synthesis process.

To further elucidate the chemical state of silver species in the composites, X-ray photoelectron spectroscopy (XPS) analysis was performed on the Ag(0)@PBPAF-2 sample. As shown in Figure 2c, the Ag 3d spectrum exhibits two distinct peaks at binding energies of 368.7 eV and 374.2 eV, which are assigned to Ag 3d_5_/_2_ and Ag 3d_3_/_2_ of metallic silver (Ag(0)), respectively [43]. The absence of other silver species signals confirms that Ag nanoparticles exist exclusively in the zero-valent state, which is consistent with the FT-IR results discussed earlier.

**Figure 3:**
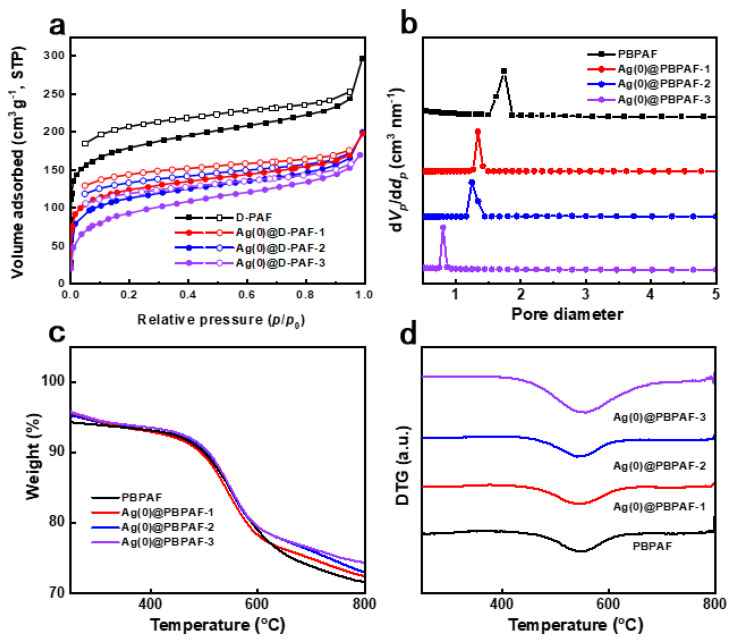
(**a**) N_2_ adsorption–desorption isotherms and (**b**) pore size distributions of synthesized samples, (**c**) TG and (**d**) DTG plots of PBPAF and Ag(0)@PBPAF samples.

Subsequently, the textural properties of the synthesized materials were investigated by N_2_ adsorption–desorption measurements at 77 K (Figure 3a,b). All samples exhibit type-I isotherms according to the IUPAC classification, characterized by rapid N_2_ uptake at low relative pressure (P/P_0_ < 0.01), indicating the predominant microporous structure. Notably, after the incorporation of Ag nanoparticles, both the specific surface area and pore volume of the Ag(0)@PBPAF composites decrease compared to the pristine PBPAF. This reduction becomes more pronounced with increasing Ag loading, suggesting partial pore blockage by the deposited nanoparticles. Furthermore, this observation is corroborated by the gradual decrease in the average pore size of the composites. The detailed textual parameters, including BET surface area, pore volume, and pore size, are summarized in Table 1. Complementarily, the actual Ag contents in Ag(0)@PBPAF-1, Ag(0)@PBPAF-2, and Ag(0)@PBPAF-3 were quantitatively determined by ICP-MS analysis to be 3.85%, 7.88%, and 10.21 wt%, respectively (Table 1), confirming the successful control over Ag loading during synthesis. To gain further insight into the stability of the composite materials, the thermal stability of the samples was evaluated by thermogravimetric analysis (TGA) under air atmosphere (Figure 3c,d). All materials show an initial weight loss below 200 °C, attributable to the removal of residual solvents and moisture. The pristine PBPAF exhibits a major weight loss between 400 and 600 °C, corresponding to the decomposition of the organic framework. Importantly, the Ag(0)@PBPAF composites demonstrate a similar decomposition temperature range, indicating that the incorporation of Ag nanoparticles does not compromise the thermal stability of the PBPAF matrix. Moreover, due to the thermal stability of the metallic Ag nanoparticles, the residual mass after combustion increases progressively with higher Ag loading, providing additional evidence for the successful and controlled incorporation of Ag nanoparticles in the composites. Collectively, these structural and chemical analyses confirm the successful fabrication of Ag(0)@PBPAF composites with well-defined metallic silver species and tailored porosity.

### 2.2. Photothermal Performance of Ag(0)@PBPAF Composites

**Figure 4:**
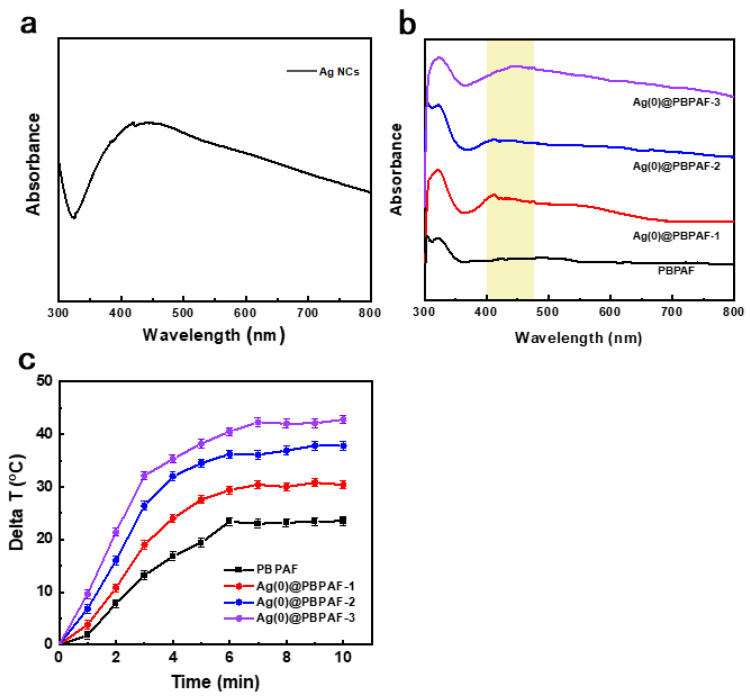
UV-Vis plots of (**a**) Ag nanoparticles, (**b**) PBPAF and Ag(0)@PBPAF composites; (**c**) Photothermal performance plots of PBPAF and Ag(0)@PBPAF composites.

The photothermal properties of the synthesized materials were systematically investigated to evaluate their potential for light-induced thermal response, which is crucial for achieving efficient desorption through photothermal conversion [39,44,45,46]. Initially, UV–visible spectroscopy was employed to analyze the optical absorption characteristics of both the pristine materials and their composites. As depicted in Figure 4a, pure Ag nanoparticles obtained from the photoreduction synthesis exhibit a discernible but broad absorption band spanning the range of 400–500 nm, which is indicative of the localized surface plasmon resonance (LSPR) phenomenon. The breadth of the band suggests a degree of polydispersity in nanoparticle size and shape, which is consistent with the mild, surfactant-free synthesis conditions employed [47]. This distinct absorption profile confirms their inherent capability to interact strongly with visible light and convert photon energy effectively. Meanwhile, the pristine PBPAF material demonstrates a fundamentally different absorption pattern, showing a distinct absorption edge primarily in the 300–350 nm range (Figure 4b), which can be rationally attributed to the π-π* transitions within its conjugated aromatic framework. This ultraviolet-dominated absorption characteristic inherently limits its photothermal conversion capability under visible light illumination.

Most notably, after the incorporation of Ag NPs, the Ag(0)@PBPAF composite spectra show a broadening and a noticeable broad feature in the 400–500 nm region, consistent with the presence of Ag NPs. The increasing intensity of this spectral feature with higher Ag loading (from Ag(0)@PBPAF-1 to Ag(0)@PBPAF-3) confirms the progressive incorporation of photothermal-active species. We acknowledge that the LSPR signature in the composites, particularly for Ag(0)@PBPAF-2, is not sharp, which can be attributed to the polydisperse nature of the Ag NPs, their potential aggregation within the pores, and strong light scattering by the porous PAF matrix. This tailored optical absorption behavior establishes a solid foundation for the subsequent photothermal performance evaluation. To further investigate the optical stability and reversibility of the photothermal process, the UV-Vis spectrum of Ag(0)@PBPAF-3 was measured after a complete adsorption–desorption cycle involving visible light irradiation. As shown in Figure S1, the spectrum recorded post-cycle is virtually superimposable with that of fresh material. The characteristic LSPR band centered around 400–450 nm remains unchanged in both position and shape. This observation provides direct evidence that the photothermal conversion driven by the LSPR effect does not induce permanent alterations to the optical properties or the chemical state of the Ag NPs. This spectral stability underpins the excellent recyclability of the adsorbent, confirming that the Ag NPs retain their structural and functional integrity as both adsorption and photothermal sites throughout the regeneration cycles.

To quantitatively evaluate the practical photothermal conversion capability, we subsequently monitored the temperature evolution profiles of different samples under standardized visible light irradiation (420–780 nm) using high-precision infrared thermal imaging. As systematically documented in Figure 4c, the temperature trajectories revealed substantial differences among the various materials. The pristine PBPAF material exhibited a relatively modest temperature increase of approximately 24 °C after 10 min of continuous illumination, which can be primarily attributed to the baseline thermal effects generated by the xenon lamp setup itself, rather than any intrinsic photothermal conversion capability of the material. In striking contrast, all Ag(0)@PBPAF composites demonstrated dramatically enhanced photothermal performance under identical experimental conditions. Specifically, Ag(0)@PBPAF-3, with the highest Ag loading, achieved a remarkable temperature elevation of 45 °C within the same duration, representing an 87.5% enhancement compared to the pristine PBPAF. Even Ag(0)@PBPAF-1, with the lowest Ag content, showed a substantial temperature increase of 30 °C, confirming that even minimal Ag incorporation significantly boosts photothermal conversion. Most importantly, the temperature rise profiles exhibited a clear dependency on irradiation time, with rapid heating observed within the initial minutes followed by gradual stabilization, indicating efficient light-to-heat conversion kinetics. The temperature enhancement also demonstrated a strong positive correlation with Ag loading content. To quantitatively assess the light-to-heat conversion capability, the photothermal conversion efficiency (η) of Ag(0)@PBPAF-3 was calculated. Following the method detailed in Supporting Information Section S1, the heat transfer coefficient (hS) was derived from the cooling curve (Figure S2). Using the steady-state temperature rise (ΔT = 45 °C), the sample absorbance at 450 nm (A = 0.89), and the incident power (I = 800 mW), the efficiency η was determined to be 32.7%. This quantitative metric confirms the high efficacy of Ag NPs in the composite for converting visible light into thermal energy. An uncertainty analysis was conducted considering the primary error sources: the calibrated power meter accuracy (±1.9%), the thermal camera temperature resolution (±0.5 °C), and sample packing variability. The error bars in Figure 4c represent the standard deviation from triplicate measurements. The reported efficiency value of 32.7 ± 1.5% encompasses this propagated uncertainty, underscoring the reliability of the measurement.

The underlying mechanism for this superior photothermal performance can be predominantly attributed to the efficient LSPR effect of the well-dispersed Ag nanoparticles within the porous matrix. When illuminated with visible light, the coherent oscillation of conduction electrons in Ag nanoparticles resonantly absorbs photons and rapidly converts them into thermal energy through non-radiative relaxation processes. The homogeneous distribution of Ag nanoparticles throughout the PBPAF, as confirmed by our earlier characterization results, ensures optimal light harvesting and minimizes scattering losses. Additionally, the porous structure of PBPAF facilitates multiple internal reflections of incident light, thereby enhancing the overall light absorption efficiency.

From an application perspective, this prominent photothermal effect presents significant potential for various applications requiring precise thermal management, particularly in photothermal-catalyzed desorption processes where localized heating can trigger controlled release of adsorbed species. The efficient and rapid temperature response, combined with the spatial and temporal controllability offered by light irradiation, positions these Ag(0)@PBPAF composites as promising candidates for developing next-generation smart adsorption systems with energy-efficient regeneration capabilities. The demonstrated photothermal performance, coupled with the previously confirmed structural stability, provides a comprehensive foundation for their practical implementation in advanced separation processes, particularly in the field of adsorptive desulfurization, where controlled regeneration is paramount for economic viability.

### 2.3. Adsorptive Desulfurization Performance of Ag(0)@PBPAF

**Figure 5:**
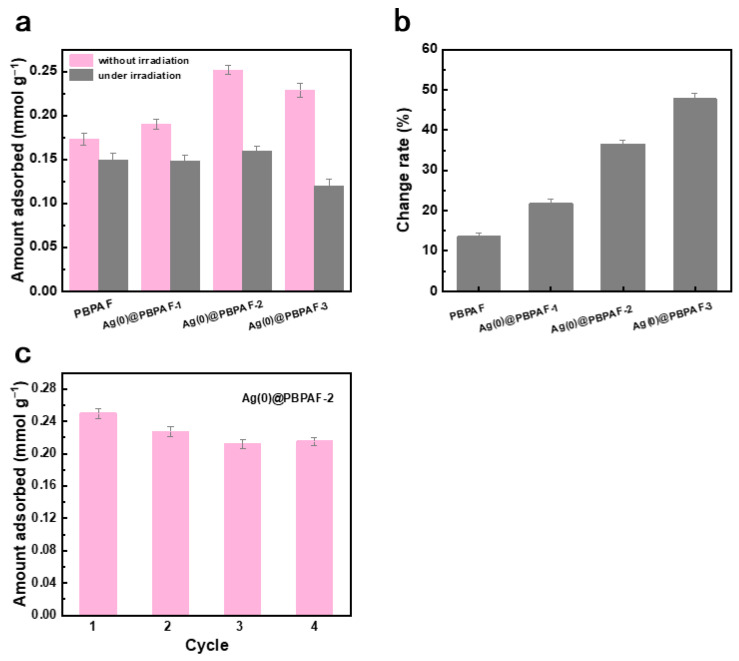
(**a**) Static adsorption test of all samples in 550 ppm BT solution, (**b**) desorption rate in the presence of light, (**c**) cyclic adsorption performance of Ag(0)@PBPAF-2. Error bars represent the standard deviation from triplicate measurements.

The desulfurization capabilities of the synthesized Ag(0)@PBPAF composites were systematically evaluated through a series of adsorption experiments. Initial static adsorption tests were conducted using a model fuel containing 550 ppm benzothiophene (BT) in isooctane to assess the fundamental adsorption performance. As illustrated in Figure 5a, the pristine PBPAF material demonstrated a moderate saturation adsorption capacity of 0.17 mmol·g^−1^ at ambient temperature, which establishes a baseline for the inherent adsorption characteristics of the porous framework. Notably, the incorporation of Ag nanoparticles resulted in substantial improvements in adsorption performance across all composite materials. Among these, Ag(0)@PBPAF-2 exhibited the most promising performance with a maximum adsorption capacity of 0.25 mmol·g^−1^, representing an approximate 47% enhancement compared to the unmodified PBPAF. This significant improvement can be rationally attributed to the establishment of specific weak interactions between the incorporated Ag nanoparticles and sulfur atoms present in the thiophenic rings of BT molecules. We have added a detailed comparison with the existing literature to objectively position the performance of Ag(0)@PBPAF-2. For instance, studies on bimetal ion-exchange zeolites, a common class of adsorbents, show varying capacities. AgCeY zeolite was reported to exhibit equilibrium adsorption amounts of 0.184 mmol·g^−1^ for thiophene (TP) and 0.301 mmol·g^−1^ for BT at 50 °C. Beyond zeolites, performance data from metal–organic frameworks (MOFs) provide further benchmarks. The adsorption capacity of pure MIL-101-NH_2_ for BT was reported to be 0.16 mmol·g^−1^. Furthermore, a silver-modified mesoporous silica material, Ag-MSN, demonstrated a high BT adsorption capacity of 0.40 mmol·g^−1^. This comparison demonstrates that the adsorption capacity achieved in this work is on par with that of various typical adsorbents reported in the literature [40,48]. The embedded Ag NPs serve a dual purpose: they act as specific adsorption sites through weak Ag–S interactions, and simultaneously, under light irradiation, they function as efficient nanoscale heaters via the LSPR effect. This design enables the unique photothermal regeneration capability. However, further increasing the Ag loading to produce Ag(0)@PBPAF-3 resulted in a discernible reduction in adsorption capacity, despite its higher metal content. This phenomenon can be explained by considering the textural properties previously discussed, where excessive Ag nanoparticle loading leads to partial pore blockage and reduced accessibility to active sites, thereby diminishing the overall adsorption efficiency. This observation highlights the existence of an optimal Ag loading level that maximizes the number of accessible active sites while maintaining sufficient porosity for efficient molecular transport.

The light-triggered regeneration capability of the adsorbents was subsequently investigated under visible light irradiation (420–780 nm). As presented in Figure 5b, the pristine PBPAF material showed minimal desorption capacity (0.02 mmol·g^−1^) under illumination, primarily attributable to the mild thermal effects generated by the xenon lamp itself. In striking contrast, all Ag(0)@PBPAF composites demonstrated significantly enhanced desorption performance under identical conditions. Particularly impressive was Ag(0)@PBPAF-3, which achieved a desorption capacity of 0.11 mmol·g^−1^, corresponding to a remarkable desorption efficiency of 48%. This superior performance unequivocally demonstrates the dual functionality of Ag nanoparticles within the composite system: serving as specific adsorption sites in the absence of light and transforming into efficient nanoscale heaters under illumination through the LSPR effect, thereby enabling precise, energy-efficient control over the adsorption–desorption process.

To evaluate the practical recyclability of the photothermal adsorbent, five consecutive adsorption–desorption cycles were conducted using Ag(0)@PBPAF-2. As shown in Figure 5c, the adsorbent retained 86% of its initial adsorption capacity after four cycles, demonstrating excellent operational stability and recyclability. The high retention of adsorption capacity strongly suggests that the composite’s structure and active Ag(0) sites remain largely intact throughout the repeated photothermal regeneration process.

**Figure 6:**
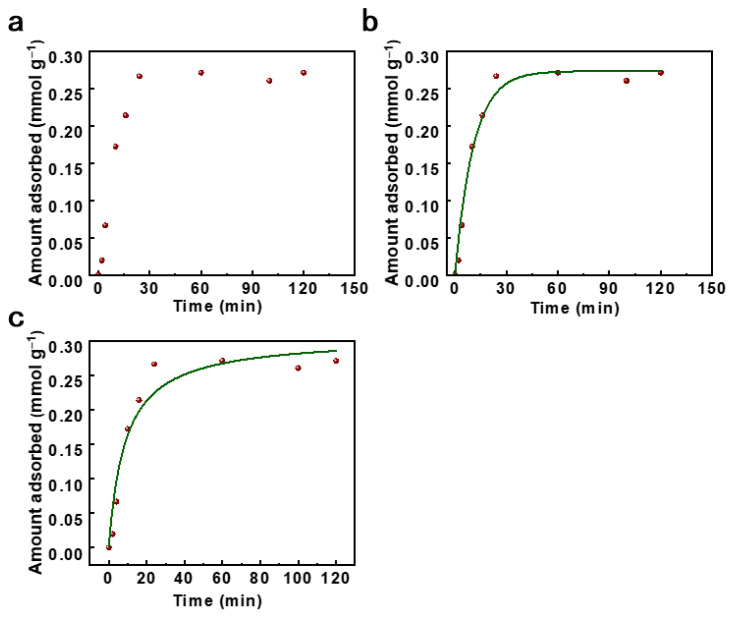
Adsorption kinetic curves of Ag(0)@PBPAF-2, (**a**) experimental results, (**b**) first-order kinetic equation fit, (**c**) second-order kinetic equation fit.

To gain deeper insights into the adsorption mechanism and rate-limiting steps, the kinetic behavior of the optimal Ag(0)@PBPAF-2 composite was thoroughly investigated. The experimental data fitted with both pseudo-first-order and pseudo-second-order kinetic models (Figure 6 and Table 2). The adsorption process exhibited characteristic rapid initial uptake, reaching equilibrium within approximately 30 min. Statistical analysis revealed that the pseudo-first-order model provided a superior fit to the experimental data (R^2^ = 0.98) compared to the pseudo-second-order model (R^2^ = 0.94), suggesting that physical interactions, including van der Waals forces and π-π interactions, likely dominate the adsorption process rather than chemical bonding.

**Figure 7:**
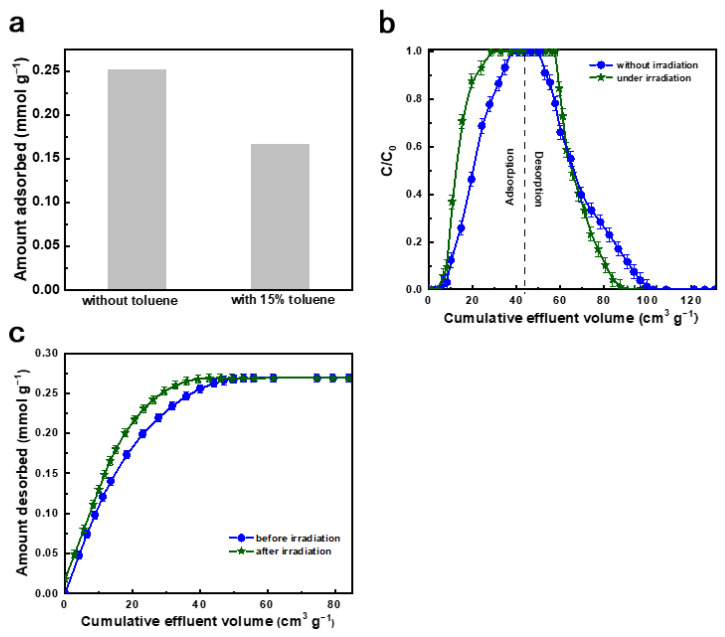
(**a**) Competitive adsorption effect diagram of Ag(0)@PBPAF-2. (**b**) Dynamic penetration curves, (**c**) desorption curves of Ag(0)@PBPAF-2 on a fixed bed with/without light exposure.

Considering practical application requirements, where real fuel streams contain various competing aromatic compounds, competitive adsorption experiments were conducted using a model fuel incorporating 15 vol% toluene as a representative aromatic competitor (Figure 7a). Remarkably, Ag(0)@PBPAF-2 maintained a substantial adsorption capacity of 0.17 mmol·g^−1^ despite the presence of this strong competitor. The calculated relative adsorption capacity retention is approximately 68% in the presence of 15 vol% toluene. This demonstrates a preliminary but distinct selectivity towards sulfur compounds over a common aromatic competitor (toluene). This preferential adsorption can be attributed to the specific affinity between Ag nanoparticles and sulfur atoms, which effectively differentiates sulfur-containing compounds from other aromatic molecules through stronger interaction energies.

Finally, to validate the industrial feasibility of the photothermal regeneration strategy, dynamic fixed-bed adsorption–desorption experiments were conducted using Ag(0)@PBPAF-2 (Figure 7b,c). During the adsorption phase, interestingly, light irradiation was observed to shorten the breakthrough time, suggesting that the locally generated heat somewhat inhibited the adsorption process, likely due to the exothermic nature of adsorption. However, during the regeneration phase, when the model fuel was switched to pure isooctane, light irradiation dramatically accelerated the desorption rate and significantly reduced the regeneration time compared to the non-illuminated case. The relationship between desorption time and desorption quantity (Figure 7c) clearly demonstrates that light irradiation achieves comparable desorption capacity in substantially less time, highlighting the energy efficiency of the photothermal regeneration process. A quantitative comparison of energy consumption further underscores the advantage of the photothermal process. As estimated in Supporting Information Section S2, the energy required for light-triggered regeneration is only a fraction (approximately 18–25%) of that needed for conventional thermal regeneration to achieve a similar desorption effect, primarily due to the highly efficient and localized heating nature of the LSPR effect. These compelling results confirm the practical viability of using light as a precise, energy-efficient trigger for controlled desorption in dynamic operating conditions, underscoring the significant potential of Ag(0)@PBPAF composites for advanced, energy-efficient desulfurization processes in practical applications. This comprehensive evaluation of adsorption performance, combined with the previously demonstrated structural and photothermal properties, establishes Ag(0)@PBPAF composites as promising intelligent adsorbents capable of efficient desulfurization combined with energy-saving regeneration through renewable solar energy.

### 2.4. Discussion on Adsorption Mechanism and Future Perspectives

The significant enhancement in adsorption capacity for benzothiophene (Figure 5a) and the high selectivity maintained in the presence of toluene (Figure 7a) strongly suggest the existence of specific interactions between the Ag nanoparticles and the sulfur atom in the thiophenic ring. While direct surface spectroscopic evidence is not provided in this study, this observation is consistent with the well-established consensus on Ag–S interactions reported across diverse fields, including adsorptive desulfurization, theoretical computation, and coordination chemistry. In the field of separation science, the specific capture of sulfur compounds by Ag-based materials is commonly attributed to two mechanisms: π-complexation between the aromatic ring and Ag and more direct S–Ag bonding. For instance, systematic studies on Ag-based adsorbents have explicitly identified direct S–Ag bonding as a crucial pathway alongside π-complexation [49]. Theoretical investigations further elucidate the electronic nature of such interactions, where the lone pair electrons on the sulfur atom coordinate with the conduction band (sp-band) of Ag, forming a weak, polar coordination bond with an energy between physisorption and strong chemisorption. This characteristic perfectly explains the high adsorption capacity coupled with excellent reversibility, enabled by photothermal triggering, observed in our material [50]. Furthermore, structural analyses of silver–thiolate complexes have confirmed a typical Ag–S bond length of approximately 2.45 Å, providing solid experimental parameters for such interactions from a structural chemistry perspective [51].

Based on this established body of knowledge, the enhanced performance of Ag(0)@PBPAF in this work is rationally attributed to the formation of weak Ag–S coordination between the Ag NPs and BT molecules. However, we fully recognize that real fuel systems constitute a complex environment with multiple coexisting components, where the adsorption process involves the synergy and competition of various interactions (e.g., solvent effects, competitive π-complexation from different aromatics). The current study, focusing on a single model compound, has limitations in elucidating the microscopic mechanism under realistically complex conditions. Meanwhile, future work will systematically investigate the adsorption performance and mechanistic evolution in the presence of a broader range of competitive species (e.g., different aromatic cores, nitrogen-containing compounds, and other sulfur homologues) to address the complex systems engineering challenge of real fuel desulfurization.

## 3. Materials and Methods

### 3.1. Synthesis of Materials

The PBPAF material was synthesized via a Suzuki coupling reaction. Typically, tetrabromotetraphenylmethane (0.39 mmol)(Alfa Aesar, Shanghai, China) and 1,4-phenylenediboronic acid (0.78 mmol) (Sigma-Aldrich, Shanghai, China) were dissolved in anhydrous DMF (Sinopharm Chemical Reagent Co., Ltd., Shanghai, China) under nitrogen atmosphere. Tetrakis(triphenylphosphine)palladium (50 mg, 43 μmol) (Alfa Aesar, Shanghai, China) and a 2 M K_2_CO_3_ aqueous solution (prepared using K_2_CO_3_ [Sinopharm Chemical Reagent Co., Ltd., Shanghai, China] and deionized water) were added as the catalyst and base, respectively. The reaction proceeded at 150 °C for 24 h. The resulting crude product was collected by filtration, washed sequentially with THF (Sinopharm Chemical Reagent Co., Ltd., Shanghai, China), chloroform (Sinopharm Chemical Reagent Co., Ltd., Shanghai, China), and deionized water, and further purified by Soxhlet extraction with THF for 24 h. The final gray-black PBPAF powder was obtained after drying at 60 °C under vacuum.

The Ag(0)@PBPAF composites were prepared by a green photoreduction method. First, the as-synthesized PBPAF was activated at 150 °C under vacuum for 24 h. Then, 100 mg of the activated support was dispersed in anhydrous methanol. A specified amount of AgNO_3_ (8, 15, or 20 mg) in methanol was added dropwise under dark conditions, and the mixture was stirred for 4 h. The resulting suspension was irradiated for 1 h using a xenon lamp equipped with a 420–780 nm filter. The final products, denoted as Ag(0)@PBPAF-1, Ag(0)@PBPAF-2, and Ag(0)@PBPAF-3, were collected by filtration, thoroughly washed, and dried at 60 °C under vacuum.

### 3.2. Materials Characterization

The synthesized materials were characterized using multiple analytical techniques. Powder X-ray diffraction (XRD) patterns were recorded on a Bruker D8 Advance diffractometer (Bruker, Billerica, MA, USA) with Cu Kα radiation over the 2θ range of 5–80°. Fourier transform infrared (FT-IR) spectra were obtained using a NICOLET NEXUS-670 spectrometer (Thermon Scientific, Waltham, MA, USA) with KBr pellets. Morphological features were examined by field-emission scanning electron microscopy (SEM) on a Hitachi Regulus 8100 microscope (Hitachi, Tokyo, Japan). Nitrogen physisorption measurements at 77 K were performed on a Micromeritics ASAP 2020 analyzer (Micromeritics Instrument Corporation, Norcross, GA, USA) to determine textural properties. Thermal stability was evaluated by thermogravimetric analysis using a NETZSCH STA 449 F5 instrument (NETZSCH-Gerätebau GmbH, Selb, Germany). UV–visible spectra were collected on a PerkinElmer Lambda 35 spectrophotometer (PerkinElmer, Waltham, MA, USA). The photothermal performance was evaluated under irradiation from a 300 W xenon lamp (CEL-HXF300, Beijing China Education Au-light Technology Co., Ltd., Beijing, China) equipped with a 420–780 nm bandpass filter. The power density at the sample position was calibrated to 800 ± 15 mW cm^−2^ using a standard optical power meter (Thorlabs PM100D with S310C sensor, Newton, NJ, USA). The temperature was monitored in real-time using a FLIR E6 infrared thermal camera (FLIR, Shanghai, China). For each measurement, 10 mg of the adsorbent powder was pressed into a pellet of uniform thickness to ensure consistent mass and surface exposure.

### 3.3. Adsorption Measurements

The model fuel was prepared by dissolving benzothiophene (BT) in isooctane at a concentration of 550 ppm. For batch adsorption tests, 50 mg of the adsorbent was mixed with the model fuel and agitated for 1 h under both dark and light irradiation conditions. The sulfur concentration in the treated fuel was quantified using an Agilent 7890A gas chromatograph (Santa Clara, CA, USA) equipped with a flame photometric detector (GC-FID). Competitive adsorption experiments were conducted by introducing toluene (15 vol%) into the 550 ppm model fuel. For dynamic adsorption–desorption evaluation, a fixed-bed reactor (inner diameter: 3 mm, length: 200 mm) was employed. Approximately 150 mg of the adsorbent was packed in the middle of the reactor, with both ends filled with quartz sand for support. The model fuel (550 ppm BT in isooctane) was fed through the bed at a flow rate of 3 mL/h. After adsorption equilibrium was reached, the feed was switched to pure isooctane. To investigate the photothermal desorption performance, the adsorbent in the quartz column was irradiated laterally with visible light, and the behavior was monitored. All batch adsorption and desorption experiments were conducted in triplicate, and reported data points represent the mean values with error bars indicating the standard deviation.

## 4. Conclusions

In this study, a series of photothermal adsorbents with varying silver contents, denoted as Ag(0)@PBPAF-n (n = 1, 2, 3), were successfully synthesized by incorporating Ag NPs into a porous aromatic framework (PBPAF) via a mild and green photoreduction method. Systematic investigation confirmed that the resulting composites exhibit remarkable photothermal properties and adsorption capacity for desulfurization. Leveraging the LSPR effect of Ag NPs, Ag(0)@PBPAF-3 achieved a rapid temperature increase of 21 °C within 10 min of visible light irradiation, demonstrating efficient photothermal conversion. Regarding adsorption performance, Ag NPs served as effective adsorption sites, exhibiting specific interactions with thiophenic sulfur compounds. This resulted in a maximum saturation adsorption capacity of 0.25 mmol·g^−1^ for thiophene on Ag(0)@PBPAF-2. More significantly, during the desorption phase, the Ag NPs functioned as nanoscale heaters under light excitation, enabling in situ and precise heating of the adsorption sites to efficiently trigger the release of adsorbed sulfur species. A desorption efficiency of up to 48% was achieved for Ag(0)@PBPAF-3. Fixed-bed adsorption experiments further validated the technical advantages of this photothermal adsorbent. Compared to conventional solvent-assisted desorption, the photothermal desorption process demonstrated a notably faster desorption rate and a shorter regeneration cycle, highlighting its significant potential for reducing energy consumption and enhancing process efficiency. This work provides a novel design strategy and practical foundation for developing next-generation intelligent and energy-efficient adsorption separation materials.

## Figures and Tables

**Table 1:** Physical parameters of synthetic materials.

Sample	S_BET_ (m^2^·g^−1^)	*V*_p_ (cm^3^·g^−1^)	Ag (wt%)
PBPAF	616	0.63	-
Ag(0)@PBPAF-1	444	0.32	3.85
Ag(0)@PBPAF-2	400	0.31	7.88
Ag(0)@PBPAF-3	327	0.30	10.21

**Table 2:** Summary of parameters fitted using pseudo-first-order and pseudo-second-order equations.

Sample	Pseudo-First-Order Model			Pseudo-Second-Order Model		
	*Q_e_* (mmol·g^−1^)	*k*_1_ (min^−1^)	R^2^	*Q_e_* (mmol·g^−1^)	*k*_2_ (min^−1^)	R^2^
Ag(0)@PBPAF-2	0.27	0.09	0.98	0.28	0.37	0.94

## Data Availability

Data is contained within the article.

## Supplementary Materials

The following supporting information can be downloaded at https://www.mdpi.com/article/10.3390/molecules31020248/s1, Figure S1. UV-Vis plots of Ag(0)@PBPAF-3 composite after light irradiation. Figure S2. Cooling curve of Ag(0)@PBPAF-3 after turning off the light irradiation.
