# Supplementary material for: Design of a Recyclable Photoresponsive Adsorbent via Green Synthesis of Ag Nanoparticles in Porous Aromatic Frameworks for Low-Energy Desulfurization
Source: Molecules. 2026 Jan 12;31(2):248. doi: 10.3390/molecules31020248 (PMC12844071; doi:10.3390/molecules31020248)
Supplement: Supplementary file 1 [file molecules-31-00248-s001.zip › molecules-4049981-supplementary.pdf]

Supporting Information

Design of a Recyclable Photoresponsive Adsorbent via Green Synthesis of Ag Nanoparticles in Porous Aromatic Frameworks for Low-Energy Desulfurization

Tiantian Li [1,*], Xiaowen Li [1], Hao Wu [1] and Qunyu Chen [1]

[1] School of Chemical Engineering and Technology, Xuzhou College of Industrial Technology, Xuzhou 221140, China

*Correspondence: litt@mail.xzcit.cn (T.L.)

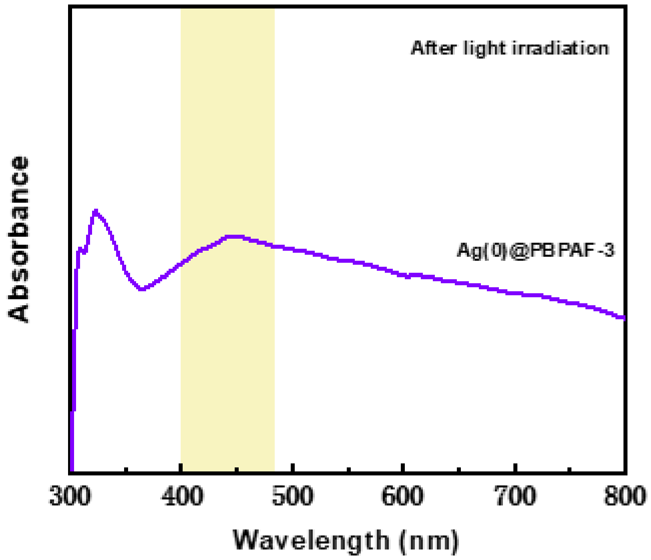

Figure S1. UV-Vis plots of Ag(0)@PBPAF-3 composite after light irradiation.

S1. Calculation of Photothermal Conversion Efficiency.

The equation for the system's heat transfer: $\sum_i m_i C_i$ (dT/dt) = $Q_{np} + Q_{sol} - Q_{loss}$, where $Q_{np} = I(1 - 10^{-A})\eta$.

The method to obtain the heat transfer coefficient (hS) from the cooling curve (new Figure S2, see below). The cooling curve data presented in Figure S2 includes inherent instrumental noise associated with infrared temperature measurement, which accounts for the minor deviations from a perfect exponential fit. The fitted time constant (τ) and

calculated photothermal conversion efficiency (η) are derived from this raw experimental data.

The final formula used: $\eta = [hS\ (T_{max} - T_{surr}) - Q_{sol}] / I\ (1 - 10^{-A})$.

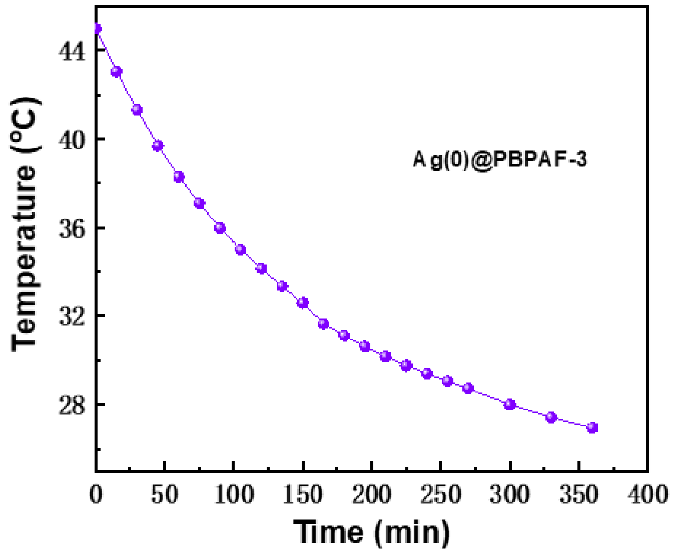

Figure S2. Cooling curve of Ag(0)@PBPAF-3 after turning off the light irradiation.

S2. Estimation of Energy Consumption for Regeneration.

Key Assumptions and Result:

Photothermal Regeneration: Energy input is the electrical energy consumed by the xenon lamp during the effective light-triggered desorption period observed in our fixed-bed experiment.

Conventional Thermal Regeneration: Energy input is the heating energy required to raise the entire adsorbent bed and react to a typical desorption temperature.

Calculation Result: Based on this model, the estimated energy consumption for the photothermal process is only about 18-25% of that required for conventional thermal heating to achieve comparable desorption, highlighting the dramatic energy savings of localized photothermal heating.
